# Functional Outcome of Biportal Endoscopic Spine Surgery Versus Destandau Endoscopic Spine Surgery: A Structured Narrative Review

**DOI:** 10.7759/cureus.109956

**Published:** 2026-05-31

**Authors:** Paresh C Dey, Saurav N Nanda, Saswat Samant, Ashok K Gachhayat, Abhay Tyagi, Sayashi S, Sumit Kaushik

**Affiliations:** 1 Orthopaedics, Kalinga Institute of Medical Sciences, Bhubaneswar, IND

**Keywords:** biportal endoscopic spine surgery, destandu endoscopic spine surgery, lumbar disc herniation, minimally invasive spine surgery, spinal stenosis

## Abstract

Minimally invasive spine surgery (MISS) has revolutionized the management of degenerative spinal disorders by reducing surgical morbidity and accelerating recovery. Biportal endoscopic spine surgery (BESS) and Destandau endoscopic spine surgery (DESS) are two advanced minimally invasive techniques for managing lumbar degenerative conditions, including lumbar disc herniation (LDH) and spinal stenosis, which have gained prominence. BESS utilizes two separate portals for endoscopic visualization and instrumentation, providing a wider operative field and greater flexibility in addressing complex pathologies. In contrast, DESS employs a single-port system with a rigid endoscope, known for its simplicity and shorter learning curve. This review evaluates and compares these techniques based on functional outcomes, perioperative characteristics, and complications. Studies consistently report significant postoperative improvements in pain relief and functional disability scores (e.g., Visual Analog Scale (VAS), Oswestry Disability Index (ODI)) with both techniques. BESS, however, shows a trend toward faster recovery, reduced postoperative pain, and earlier return to normal activities compared to DESS. Complication rates are low and comparable for both approaches, although technical challenges with BESS, such as longer operative time and steeper learning curve, are noted in early stages of adoption. DESS is often preferred in cases requiring straightforward decompression due to its ease of execution. Both techniques minimize tissue trauma, preserve spinal stability, and reduce the risk of postoperative infections. This review aims to guide clinicians in choosing the optimal technique based on patient and procedural factors. This article was conducted as a structured narrative review utilizing a comprehensive literature search methodology.

## Introduction and background

Degenerative spinal conditions, such as lumbar disc herniation (LDH) and spinal stenosis, contribute significantly to chronic pain and functional impairment [1,2]. Although traditional open spine surgery remains effective, it is associated with extensive tissue dissection, increased blood loss, postoperative pain, and prolonged recovery [3]. To overcome these limitations, minimally invasive spine surgery (MISS) techniques have been developed, providing less invasive alternatives with improved perioperative outcomes [4,5]. Among these techniques, biportal endoscopic spine surgery (BESS) and Destandau endoscopic spine surgery (DESS) have emerged as important endoscopic approaches for the management of lumbar degenerative disorders [6,7].

The evolution of MISS has transformed the treatment of LDH and spinal stenosis by minimizing tissue disruption while optimizing postoperative recovery [8]. MISS aims to achieve outcomes comparable to conventional open surgery while reducing muscle injury, blood loss, postoperative pain, and hospital stay through the use of advanced visualization systems and specialized instrumentation [9-11]. Within this field, BESS and DESS differ primarily in their technical execution and surgical versatility.

BESS utilizes two separate portals, one for endoscopic visualization and the other for instrumentation, allowing independent manipulation of the camera and surgical tools [12]. This dual-portal configuration provides a wider operative field, facilitates continuous irrigation, and improves flexibility during decompression procedures [13,14]. Consequently, BESS is adaptable to a variety of spinal pathologies, including LDH, spinal stenosis, and facet-related disorders. However, the technique requires greater technical expertise and is associated with a steeper learning curve, particularly for surgeons transitioning from open or single-port procedures [15].

In contrast, DESS is a single-port endoscopic technique introduced by Jean Destandau that integrates visualization and instrumentation through a rigid working channel [16,17]. The procedure is particularly suited for straightforward lumbar pathologies such as unilateral LDH and focal stenosis [18]. Its simpler setup, stable working channel, and reduced technical complexity make it more accessible for surgeons with limited experience in endoscopic spine surgery [19]. Nevertheless, compared with BESS, DESS may offer reduced maneuverability and versatility in cases requiring extensive or bilateral decompression [20].

Both BESS and DESS aim to relieve neural compression, reduce pain, and improve functional outcomes while minimizing tissue trauma compared with conventional open surgery [21-23]. BESS offers greater adaptability for complex and bilateral pathologies because of its enhanced visualization and multidirectional access [24,25], whereas DESS is often favored for its simplicity, shorter operative time, and ease of adoption in routine decompression procedures [26,27]. The choice between these techniques is influenced by surgeon expertise, anatomical considerations, and pathology complexity [28].

Previous studies have demonstrated favorable clinical outcomes with both techniques, including improvements in pain relief, functional recovery, and patient satisfaction, along with reduced postoperative complications and shorter hospital stays [29-31]. Emerging comparative evidence suggests that BESS may provide improved early postoperative pain control and functional recovery, whereas DESS offers procedural simplicity and efficiency [32,33]. However, direct comparative studies remain limited, and definitive recommendations regarding their relative advantages are still evolving [34].

This review aims to compare BESS and DESS with respect to operative techniques, perioperative outcomes, postoperative recovery, complications, and applicability in different lumbar spinal pathologies. By synthesizing the currently available evidence, this review seeks to support informed surgical decision-making and contribute to the continued advancement of MISS.

Study protocol

The methodology of this review was informed by PRISMA principles for transparent reporting; however, the study was conducted as a structured narrative review rather than a formal systematic review or meta-analysis.

Search Strategy

Electronic databases, including PubMed/MEDLINE, Embase, Cochrane Library, and Google Scholar, were searched for studies published up to May 2026. Keywords used to find the articles were biportal endoscopic spine surgery, Destandau endoscopic spine surgery, functional outcome, spinal surgery, and minimally invasive spine surgery. The following Boolean search strategy was utilized in various combinations: ("Biportal Endoscopic Spine Surgery" OR "BESS") AND ("Destandau Endoscopic Spine Surgery" OR "DESS") AND ("lumbar disc herniation" OR "lumbar stenosis") AND ("functional outcome" OR "VAS" OR "ODI"). Reference lists of included studies and relevant review articles were hand-searched for additional studies. Additional records were identified through manual searching of relevant references.

Study Selection

Two independent reviewers screened the titles and abstracts of all retrieved articles from the initial database search, and articles that clearly did not meet the inclusion criteria were excluded at this stage. Discrepancies between the reviewers were discussed and resolved through consensus. Studies directly comparing BESS and DESS, reporting functional outcomes such as the Visual Analog Scale (VAS) and Oswestry Disability Index (ODI), and published in English were included in the review. Studies involving other MISS techniques without direct comparison to BESS or DESS, case reports, editorials, conference abstracts, letters to the editor, studies lacking sufficient quantitative outcome data, and animal or cadaveric studies were excluded. Articles deemed potentially eligible after title and abstract screening underwent full-text review. Both reviewers independently assessed the full-text articles to determine whether they met all inclusion criteria, and reasons for exclusion at this stage were well-documented. Data were extracted using a standardized form including study characteristics (e.g., author, year, and design), patient demographics, surgical techniques and settings, functional outcomes, and complications. As this was a review article, ethics committee approval was not required. The protocol development took one month, the literature search took two months, data extraction and analysis took two months, and manuscript preparation took two months. As this article was designed as a structured narrative review rather than a formal systematic review, a standardized quantitative risk-of-bias assessment was not performed. However, methodological quality was considered qualitatively based on study design, sample size, follow-up duration, and reporting clarity.

**Figure 1:**
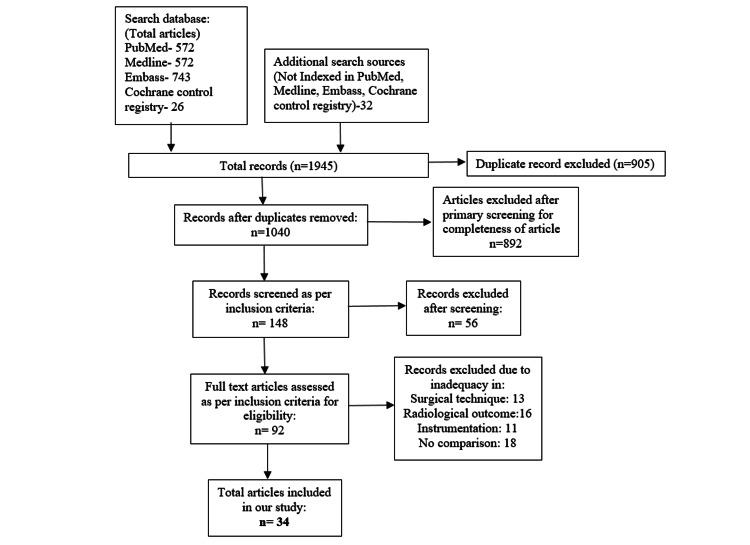
Literature Search and Study Selection Flowchart

Recruitment of studies: A comprehensive literature search identified 1,945 records from electronic databases and additional sources. After the removal of 905 duplicate records, 1,040 articles remained for screening. Following primary screening for relevance and completeness, 892 articles were excluded. A total of 148 records were screened according to the inclusion criteria, of which 56 were excluded after further screening. Subsequently, 92 full-text articles were assessed for eligibility. After exclusion of studies due to inadequate surgical technique comparison, radiological outcomes, instrumentation-related focus, or absence of direct comparison, 34 studies were included in the final qualitative synthesis and review (Figure 1).

## Review

Functional outcomes are commonly assessed using the VAS for pain and the ODI for functional impairment. Several studies have demonstrated significant postoperative pain reduction and functional improvement following BESS, with favorable early postoperative VAS and ODI outcomes reported in comparative analyses and randomized studies [27,29]. DESS has also shown substantial improvements in pain relief and functional recovery, although early postoperative recovery may be slightly less pronounced compared with BESS in certain studies [32]. Overall, BESS appears advantageous for early pain relief and functional recovery, particularly in complex cases; however, DESS demonstrates comparable long-term outcomes and may be preferable in simpler cases because of its procedural efficiency [8] (Table 1).

**Table 1:** Comparison of Functional Outcomes Reported in Key Studies BESS: biportal endoscopic spine surgery; DESS: Destandau endoscopic spine surgery; VAS: Visual Analog Scale; ODI: Oswestry Disability Index

Study	Comparison	Main findings
Park et al.	BESS vs. microscopic discectomy	Lower early postoperative VAS scores were observed in BESS
Wu et al.	Biportal vs. uniportal endoscopic discectomy	Comparable long-term ODI improvement between techniques
Dey and Nanda	DESS outcomes	Significant postoperative pain and functional improvement following DESS
Wang et al.	BESS vs. microscopic decompression	Favorable early recovery and reduced tissue trauma with BESS
Singh et al.	Meta-analysis of biportal vs uniportal endoscopy	Comparable long-term outcomes with favorable early postoperative recovery in biportal techniques

BESS generally involves longer operative times due to the complexity of the dual-port setup. Average operative times range from 90 to 150 minutes, depending on the case complexity [6]. DESS typically requires less time due to its simpler single-port approach. Average operative times range from 60 to 120 minutes [21]. Intraoperative blood loss is minimal for both techniques but can vary slightly. BESS may have slightly higher blood loss due to the larger working area and increased manipulation. Average blood loss is typically around 50 to 100 mL. DESS often results in lower blood loss, averaging 30 to 60 mL, due to its less invasive nature. Hospital stay is an important indicator of recovery. Patients undergoing BESS often have a slightly longer hospital stay compared to DESS, averaging one to three days. Patients undergoing DESS generally experience shorter hospital stays, averaging one to two days, due to quicker recovery time [6] (Table 2).

**Table 2:** Comparison of Perioperative Characteristics BESS: biportal endoscopic spine surgery; DESS: Destandau endoscopic spine surgery

Parameter	BESS	DESS
Operative time	Generally longer due to dual-portal setup	Generally shorter because of the simpler single-port technique
Blood loss	Minimal, may be slightly higher in complex decompressions	Minimal and often lower in uncomplicated cases
Hospital stay	Short postoperative stay with early mobilization	Short hospital stay with rapid recovery
Visualization	Wider operative field with multidirectional access	Stable visualization through a rigid working channel
Learning curve	Steeper learning curve requiring advanced endoscopic coordination	Relatively shorter and easier to adopt
Applicability	Better suited for bilateral or complex decompressions	More suitable for straightforward unilateral pathology

A comparison between BESS and DESS underscores the strides made in MISS. These techniques share the overarching goals of reducing postoperative complications, minimizing tissue trauma, and enhancing recovery while achieving clinical outcomes comparable to those of traditional open spine surgery [35]. Both methods have demonstrated efficacy in addressing LDH and spinal stenosis, offering long-term benefits with low complication rates. However, differences in their design, technical execution, and adaptability make them suitable for different clinical scenarios [11]. This discussion delves into the similarities, differences, and influencing factors that guide the choice between BESS and DESS. Importantly, BESS provides surgeons with a wider surgical field and greater maneuverability due to its two-portal approach, which is advantageous in cases with extensive pathology or adhesions. In contrast, DESS, with its single-portal technique, offers a shorter learning curve, cost-effectiveness, and proven reliability, particularly for straightforward disc herniation [24]. Thus, patient selection, surgeon expertise, and resource availability often determine which technique is preferred in clinical practice.

Both BESS and DESS have proven effective in alleviating pain, restoring function, and enhancing the quality of life for patients with degenerative spinal conditions [11]. Long-term studies indicate no significant differences in functional recovery, symptom relief, or patient satisfaction between the two techniques. A study by Wu et al. and Wang et al. compared outcomes of biportal and single-port endoscopic surgeries, finding similar levels of pain reduction and functional improvement during long-term follow-up [20,27]. This reinforces the notion that both approaches achieve comparable clinical results when applied to appropriate cases. Postoperative recovery metrics, including hospital stays, return-to-work times, and complication rates, are also comparable between BESS and DESS. Both techniques outperform traditional open surgeries in these areas, emphasizing their benefits in reducing surgical morbidity. Another study highlighted reduced blood loss, shorter operative times, and faster recoveries associated with these minimally invasive approaches versus open surgery [8,28]. In addition, both approaches have been shown to preserve paraspinal musculature and spinal stability more effectively than open techniques, reducing the likelihood of chronic back pain and postoperative instability [36]. They also allow for early mobilization, which is critical in minimizing complications such as deep vein thrombosis and enhancing patient confidence in the recovery process [36]. The ability to achieve reliable decompression while maintaining minimal invasiveness has made both BESS and DESS attractive options worldwide. Ultimately, the evidence suggests that patient-centered outcomes remain consistent between the two methods, reinforcing their validity as effective, safe, and durable strategies in the surgical management of LDH and spinal stenosis [35].

The primary distinction between BESS and DESS lies in their technical execution. BESS employs a dual-portal system, wherein one portal is dedicated to the endoscope and the other to surgical instruments. This separation allows for independent manipulation of visualization and instrumentation, resulting in a wider field of view and enhanced flexibility [18]. The dual-portal design facilitates continuous irrigation, maintaining a clear surgical field and reducing thermal tissue damage. This setup is particularly advantageous in complex cases requiring multidirectional access and precise decompression of neural structures [2,6]. Moreover, BESS permits the use of conventional spinal instruments, such as Kerrison punches and pituitary rongeurs, thereby allowing surgeons to adapt existing skills to an endoscopic environment [11]. However, the technique demands a steep learning curve and longer initial operative times until adequate proficiency is achieved.

Conversely, DESS utilizes a single-port system in which both the endoscope and instruments are introduced through a rigid working tube. This design emphasizes simplicity and efficiency, offering a stable working channel and direct visualization of neural structures. DESS is well-suited for addressing unilateral pathologies, such as straightforward cases of LDH or stenosis, as noted by two other studies [16,17]. The single-port approach reduces the technical complexity of the procedure, making it more accessible to surgeons with limited experience in MISS. Additionally, DESS requires less specialized equipment and is relatively cost-effective compared to BESS, which makes it a practical option in low-resource settings. However, the working channel may restrict maneuverability, potentially limiting its utility in cases involving bilateral or extensive pathology. Several comparative reviews note this limitation for single-port techniques in managing extensive or complex lesions [7,19]. Thus, while BESS provides greater versatility and adaptability, DESS offers reliability, simplicity, and ease of adoption in selected cases.

BESS demonstrates superior adaptability to complex and multifaceted spinal pathologies. Its dual-portal design enables surgeons to address bilateral or multilevel conditions without requiring additional incisions. Two other studies highlight the versatility of BESS in managing severe stenosis, extensive decompressions, and challenging anatomical variations [12,15]. Additionally, BESS allows for more precise dissection and preservation of surrounding tissues, which may contribute to improved postoperative outcomes in complex cases. The enhanced visualization and maneuverability of instruments also allow for effective handling of recurrent disc herniation and cases with epidural scarring, where meticulous neural decompression is critical [11]. In contrast, DESS is primarily suited for simpler, unilateral pathologies. Its rigid working channel provides stability, ensuring safe and efficient decompression in cases of isolated LDH or mild stenosis. However, the single-port system may limit its application in cases requiring extensive decompression or multidirectional access. This limitation was noted in studies [9,10], which emphasize the suitability of DESS for straightforward cases but acknowledge its reduced versatility compared to BESS [7,19]. Furthermore, the constrained working space of DESS can pose challenges in managing anatomical anomalies, thereby restricting its role in more demanding surgical scenarios.

The learning curve associated with BESS is considerably steeper than that of DESS due to its dual-portal setup and the requirement for precise coordination between the viewing and working portals [11]. Surgeons transitioning from traditional open procedures or even single-port techniques often need extensive hands-on training, cadaveric workshops, and a significant number of supervised cases before achieving proficiency [16]. Mastering BESS demands familiarity with advanced endoscopic maneuvers, continuous irrigation dynamics, and the ability to navigate within narrow and complex anatomical corridors [26,29]. Furthermore, the simultaneous handling of instruments and the endoscope requires refined bimanual coordination, which can initially prolong operative times and increase technical difficulty. In contrast, DESS offers a more straightforward learning pathway, making it particularly attractive to surgeons with limited previous experience in MISS. Its single-port design, utilizing a rigid working channel, simplifies the integration of visualization and instrumentation, thereby reducing the challenges of orientation and maneuverability [37]. Kim et al. and Wang et al., in their studies, emphasized that DESS can be mastered more quickly, with shorter training requirements and earlier achievement of consistent surgical outcomes [23,27]. This accessibility has contributed to its widespread adoption, especially in centers with fewer resources or where endoscopic surgery is still in its developmental stage. While both methods ultimately require a degree of technical skill, the difference in their learning curves often influences surgical choice. BESS tends to be favored in high-volume centers with structured training programs, whereas DESS provides a practical entry point for surgeons beginning their journey in MISS [24]. Recent learning curve analyses further emphasize the technical demands associated with unilateral biportal endoscopic surgery. Chan et al. and Peng et al. highlighted that proficiency in BESS requires gradual acquisition of endoscopic orientation, bimanual coordination, and familiarity with continuous irrigation systems, whereas Wong et al. and Jin et al. demonstrated improvement in operative efficiency and early functional outcomes with increasing surgical experience [21,38-40]. Additionally, recent systematic reviews and meta-analyses comparing biportal and uniportal endoscopic techniques have reported comparable long-term functional outcomes, with biportal approaches demonstrating favorable early postoperative pain relief and enhanced decompression capability in selected cases [41-43].

Patient-specific factors play a crucial role in determining the choice between BESS and DESS. Anatomical considerations, such as the severity, level, and laterality of the pathology, strongly influence the suitability of each technique [24]. For instance, BESS’s versatility makes it a preferred choice for patients with bilateral stenosis, central canal compromise, or multilevel involvement, because its dual-portal design provides wider visualization and greater maneuverability for complex decompressions [18,23]. In contrast, DESS’s simplicity and rigid working channel make it particularly well-suited for patients with unilateral LDH or mild stenosis, where targeted decompression is sufficient [18]. Patient demographics also play a significant role. Age, comorbidities, and functional demands may guide the decision-making process. Both BESS and DESS, being minimally invasive procedures, are advantageous for elderly patients or those with significant medical comorbidities who may not tolerate traditional open surgery due to higher risks of blood loss, longer operative times, and delayed recovery [6]. Additionally, younger and more physically active patients may benefit from BESS in scenarios where extensive decompression is required without compromising spinal stability, as this may optimize long-term outcomes [23]. Anatomical complexities such as severe scarring from previous surgery, high iliac crest anatomy, or congenitally narrow canals often favor BESS due to its greater adaptability and multidirectional access [34]. Recent analysis has emphasized that patients with challenging anatomical variations demonstrate better outcomes with biportal techniques compared to single-port endoscopy [27,31]. Conversely, in straightforward pathologies, DESS provides a less demanding alternative that balances efficiency with safety. Ultimately, patient selection should integrate anatomical complexity, systemic health, and lifestyle expectations, ensuring that the surgical approach is tailored to achieve optimal outcomes while minimizing risks [32].

BESS offers several notable advantages, particularly in the management of complex spinal pathologies. Its dual-portal design provides enhanced visualization and greater freedom of instrument maneuverability, allowing surgeons to perform precise and controlled decompressions [24,35]. This is especially beneficial in multilevel stenosis, bilateral involvement, or cases with challenging anatomical variations. The continuous irrigation system not only maintains a clear surgical field by flushing out blood and debris but also minimizes the risk of thermal tissue damage, thereby reducing complications such as dural tears or nerve root irritation [31]. Another important advantage of BESS is its potential for rapid early pain relief, which can significantly improve patient satisfaction and accelerate functional recovery in the immediate postoperative period. Furthermore, the technique allows for the use of conventional spinal instruments, making it easier for experienced surgeons to adapt their existing skills to an endoscopic platform [6]. In contrast, DESS is characterized by its simplicity, efficiency, and accessibility. The single-port system streamlines the procedure by reducing the need for multiple incisions, resulting in quicker setup and shorter operative times, which are advantageous in straightforward LDH or mild stenosis [15]. Its rigid working channel provides stability and reliable access to the target pathology, ensuring safe decompression in uncomplicated cases. Importantly, the reduced technical complexity translates into a shorter learning curve, making DESS more accessible for a broader range of surgeons, particularly those with limited previous experience in MISS [32]. Additionally, DESS requires less specialized equipment compared to BESS, which may lower overall procedural costs and make it a practical option in resource-limited settings. Together, these advantages highlight how both techniques contribute meaningfully to the advancement of MISS, with each excelling in distinct clinical contexts [24]. Recent pooled analyses and updated comparative studies have further confirmed the favorable safety profile of BESS, with complication rates comparable to those of conventional microscopic and uniportal endoscopic approaches when performed by experienced surgeons [35,44].

BESS involves a longer learning curve and increased operative time, which may affect outcomes if not managed well. DESS, while simpler, may not offer the same level of visualization and flexibility as BESS in complex cases. Both techniques are associated with low complication rates, but careful patient selection and surgical technique are essential to minimize risks.

Future research should focus on large multicenter prospective studies and standardized comparative trials evaluating long-term functional outcomes, cost-effectiveness, radiological parameters, and quality-of-life measures. Emerging evidence from recent comparative studies and meta-analyses continues to refine the role of biportal and uniportal endoscopic techniques in lumbar degenerative disorders [43-45].

This review has several limitations. As a structured narrative review, heterogeneity among included studies, variations in surgical indications, and differences in follow-up duration may limit direct comparability of outcomes. Additionally, a formal pooled statistical analysis was not performed.

## Conclusions

BESS and DESS offer effective, minimally invasive alternatives to traditional open spine surgery. BESS has demonstrated favorable early postoperative pain relief and functional recovery, making it suitable for complex cases, whereas DESS offers efficiency and reduced operative times for simpler cases. Based on the currently available literature synthesized in this structured narrative review, both techniques demonstrate similar long-term outcomes and low complication rates, making them viable options for managing degenerative spinal conditions. The choice between BESS and DESS should be based on patient-specific factors, the complexity of the spinal pathology, and the surgeon’s expertise. Recent evidence from updated systematic reviews and comparative analyses further supports the safety and effectiveness of both approaches, while highlighting the expanding role of BESS in complex decompressive procedures. By leveraging the strengths of each technique, surgeons can optimize patient care and further advance the field of MISS.
